# Longevity or hypoxia: who’s driving?

**DOI:** 10.18632/aging.102174

**Published:** 2019-08-12

**Authors:** Matthew E. Pamenter, Daniel Munro

**Affiliations:** 1 Department of Biology, University of Ottawa, Ottawa, ON, Canada; 2 University of Ottawa Brain and Mind Research Institute, Ottawa, ON, Canada

**Keywords:** longevity, hypoxia tolerance, mitochondria, antioxidants, hypoxic metabolic response, metabolic rate

The balance between reactive oxygen species (ROS) production and consumption within a cell is achieved by a complicated suite of enzymatic processes, which present a double-edged sword in the maintenance of cellular function. Under normal conditions, ROS are important 2^nd^ messengers that play a critical role in cellular signalling. However, under abnormal conditions – which may result from life in a challenging environment (*e.g.*, a hypoxic niche), pathophysiology or disease, or simply due to the slow accumulation of deficits in cellular function with aging – ROS production often overcomes the cellular capability to consume ROS, resulting in free radical-mediated damage to DNA, proteins, and lipids. Such derangements in ROS homeostasis have been linked broadly to a wide range of diseases and to aging, although these links are debated in many fields [1]. For example, in ischemic pathology in heart and brain, the electron transport system becomes overtly reduced in the absence of molecular oxygen, resulting in the reversal of succinate dehydrogenase and the accumulation of a large succinate pool [2]. During reperfusion, this pool of succinate is thought to be rapidly oxidized, resulting in a deleterious burst of ROS that quickly overwhelms the cells’ scavenging abilities, damages cellular components, and activates cell death pathways. Similarly, Harman proposed that damage inflicted by ROS to biomolecules gradually accumulates over a much longer time-scale, and that this accumulation underlies the progression of aging, and contributes to the eventual demise of an organism [3].

This “oxidative stress theory of aging” is one of the most widely publicized and hotly contested theories in the field of gerontology, and has been the subject of numerous experimental tests over the past several decades. A popular means of testing this theory has been to compare levels of mitochondrial ROS production between species with highly divergent lifespans using simple Amplex ultrared fluorescence assays. The results from these experiments have been equivocal. For example, a comparison between short-lived mice (lifespan 3-4 yrs.) and long-lived naked mole rats (NMRs, lifespan > 30 yrs.), failed to find a difference between species in the rate of ROS production from isolated heart mitochondria [4]. However, we have recently demonstrated that using the classic Amplex system with isolated mitochondria underestimates ROS production by as much as 80% in some tissues, and with some respiratory substrates, because it only measures H_2_O_2_ that escapes matrix antioxidants and diffuses to the reaction medium [5]. This methodological limitation calls into question previous inter-species comparisons of mitochondrial ROS generation as it pertains to aging. In a recent follow-up study we demonstrated that, although mice and NMRs in fact likely do not differ greatly in their rates of ROS production, mitochondria of the longer-lived NMR have a far greater capacity to scavenge ROS [6]. As a consequence, the matrix steady-state [H_2_O_2_] should be kept lower in mitochondria of the long-lived NMR, which is in line with a putative role for mtDNA oxidation in driving senescence [7].

These findings are of obvious importance to the field of aging as they reveal a serious flaw in a widely-used methodology, and also highlight the importance of a largely overlooked component of mitochondrial ROS management: matrix scavenging. Building upon these findings holds considerable promise for reorienting studies addressing a putative role for ROS in aging. However, these results also hint at another largely overlooked and potentially fatal problem in testing the oxidative stress theory of aging, which is the question of what is the evolutionary driving force behind putatively beneficial adaptations in the management of ROS in long-lived species?

This issue is highlighted in the study of divergent species that inhabit niches in which they experience periodic hypoxia or anoxia (*e.g.*, in poorly-ventilated underground burrows, at altitude, or under frozen ponds and streams). Given the clear role that mitochondrial ROS play in hypoxia/ischemia-reperfusion injury, it is perhaps not surprising that recent studies have described optimized ROS management capabilities in mitochondria of some of the most hypoxia- and anoxia-tolerant model organisms, including NMRs [6], freshwater turtles [8], and the longest-lived metazoan, the Atlantic clam (*Arctica islandica*, Munro and Blier, in preparation). By extension, it may not be a coincidence that these species are longer-lived than closely-related hypoxia-intolerant cousin species; improved ROS management capabilities derived from adapting to hypoxia may also confer protection against the accumulation of ROS-mediated damage to macromolecules associated with aging. If this is true, then there may be a correlation between hypoxia-tolerance and longevity that has been largely overlooked in the literature. Indeed, many long-lived species (specific to their lineage) are also hypoxia-tolerant, including NMRs, turtles, numerous bat species, *A. islandica*, goldfish, crucian carp, and sturgeon. A truly fascinating question then emerges regarding the driving force behind the evolution of beneficial ROS management: is longevity in hypoxia-tolerant species a bi-product of cellular adaptations to life in hypoxia, or is it driven by other, independent factors? Answering this question will be a complex undertaking but the answer may be pivotal to unravelling the importance of the oxidative stress theory of aging in a comparative context. Importantly, longevity can evolve without adaptation to hypoxia: humans are long-lived but are not tolerant to hypoxia. Carefully delineating which adaptations are linked to the hypoxia-tolerance, longevity, or both, may be paramount in orienting translational approaches to prevent premature aging in human – and to avoid pursuing mechanisms that have nothing to do with longevity and aging *per se*.
